# The Nucleoprotein and Phosphoprotein of Measles Virus

**DOI:** 10.3389/fmicb.2019.01832

**Published:** 2019-08-21

**Authors:** Serafima Guseva, Sigrid Milles, Martin Blackledge, Rob W. H. Ruigrok

**Affiliations:** Université Grenoble Alpes, Le Centre National de la Recherche Scientifique, Commissariatá l'Energie Atomique et aux Energies Alternatives, Institut de Biologie Structurale, Grenoble, France

**Keywords:** measles virus, nucleoprotein, phosphoprotein, Cryo-EM, NMR, X-ray crystallography, RNA binding

## Abstract

Measles virus is a negative strand virus and the genomic and antigenomic RNA binds to the nucleoprotein (N), assembling into a helical nucleocapsid. The polymerase complex comprises two proteins, the Large protein (L), that both polymerizes RNA and caps the mRNA, and the phosphoprotein (P) that co-localizes with L on the nucleocapsid. This review presents recent results about N and P, in particular concerning their intrinsically disordered domains. N is a protein of 525 residues with a 120 amino acid disordered C-terminal domain, N_tail_. The first 50 residues of N_tail_ extricate the disordered chain from the nucleocapsid, thereby loosening the otherwise rigid structure, and the C-terminus contains a linear motif that binds P. Recent results show how the 5′ end of the viral RNA binds to N within the nucleocapsid and also show that the bases at the 3′ end of the RNA are rather accessible to the viral polymerase. P is a tetramer and most of the protein is disordered; comprising 507 residues of which around 380 are disordered. The first 37 residues of P bind N, chaperoning against non-specific interaction with cellular RNA, while a second interaction site, around residue 200 also binds N. In addition, there is another interaction between C-terminal domain of P (XD) and N_tail_. These results allow us to propose a new model of how the polymerase binds to the nucleocapsid and suggests a mechanism for initiation of transcription.

## Introduction

**Figure 1:**
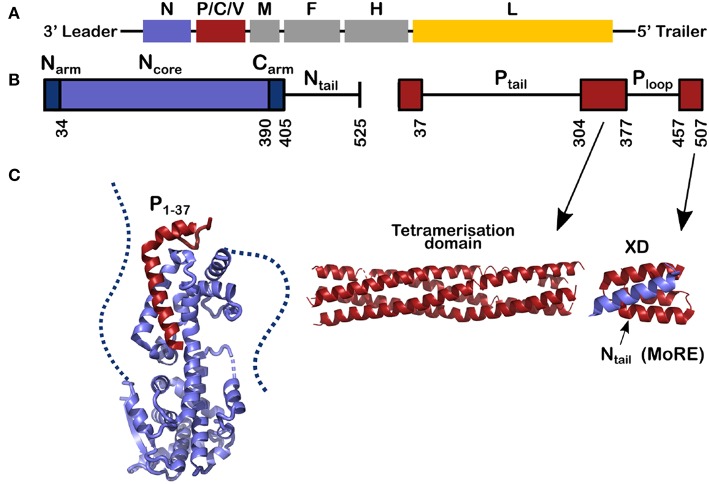
Schemes for the genes and for the nucleoprotein (N) and phosphoprotein (P) of measles virus. **(A)** The viral RNA of measles virus with its genes; The second gene codes for 3 proteins, P, C, and V and the rest of the genes code for one protein. Throughout this review the structures of N are shown in blue, for P in red and in Figure 8 the polymerase of VSV is shown in yellow. **(B)** The schemes of N and P proteins show the structured parts with colored bars and the disordered parts with a line. **(C)** The structured parts of N and P. Left, for the structure of N^0^P we used the PDB (Protein Data Bank) number 4CO6 and we show the N_arm_ (left) and the C_arm_ (right) with dash; middle, for the tetramerization domain of P we used PDB 3ZDO and right, for the complex between the linear helical motif of N_tail_ and the X domain of P we used PDB 1T6O.

Measles virus is a human virus that, although the vaccine is very efficient, still kills about 100,000 people per year (Laksono et al., 2018; WHO, 2019). Measles virus is a single-stranded negative-sense RNA virus belonging to the *Paramyxoviridae* family. The viral RNA, 15,894 nucleotides long, is bound by thousands of copies of the viral nucleoprotein, forming the helical nucleocapsid (NC). The viral polymerase complex, comprising the viral L protein (L, for Large protein) and phosphoprotein, uses the NC as the template for transcription and replication. The only known structure of L of the viruses of *Mononegavirales* order, to which also paramyxoviruses belong, is the one from vesicular stomatitis virus (VSV) (Rahmeh et al., 2010; Liang et al., 2015; Qiu et al., 2016), and several reviews have used this structure as a model for the function and structure of measles virus L (Sourimant and Plemper, 2016; Fearns and Plemper, 2017). Figure 1 shows the genes of the measles virus, coding for eight proteins within six genes: nucleoprotein (N, blue), phosphoprotein (P, red), matrix (M), the fusion protein (F), and haemagglutinin (H) (the spike proteins), and the polymerase (L, yellow). The gene of P also encodes the mRNAs for the C and V proteins. These proteins have been found in many other Paramyxoviruses and speculated to be involved in regulation of host cell immune response through different pathways. In addition, it should be mentioned that V of measles virus share the first 231 amino acids with P and thus can also maintain N chaperoning.

This review describes recent results on the phosphoprotein (P) and nucleoprotein (N) of measles virus. The classical paradigm that a protein must have a defined and stable structure and that the functional mechanisms of the protein in the cell can be derived from this structure, is not applicable to P and N. This is because most of P is disordered, as is the C-terminal tail of N (Tompa, 2002; Uversky, 2002; Karlin et al., 2003; Longhi et al., 2003; Bourhis et al., 2004; Gerard et al., 2009; Habchi et al., 2014). As shown in Figure 1, 120 residues of N and 265 + 79 residues of P are intrinsically disordered in their functional state. Together this makes up to 1392 RNA bases, almost 9% of the full genome.

## Structures and Complexes Between N and P

P and N have multi-domain structures with alternating ordered and disordered regions (Figure 1). P starts with a long disordered tail (304 amino acids) followed by a tetrameric domain of four long, parallel helices between residues 304 and 377 (Figure 1). Tetramer domains of similar length are found in Sendai, Nipah (Tarbouriech et al., 2000; Communie et al., 2013a; Bruhn et al., 2014). P of mumps also forms a tetramer of the same length but with two anti-parallel dimers (Cox et al., 2013; Pickar et al., 2015). For Sendai, Nipah and measles viruses, the polymerase binds to the tetramerization domain (Bowman et al., 1999; Bloyet et al., 2019; Bruhn et al., 2019). At the C-terminus of P there is another folded domain (XD) which is linked to the tetramerization domain via the unfolded P_loop_ (residues 377–457).

**Figure 2:**
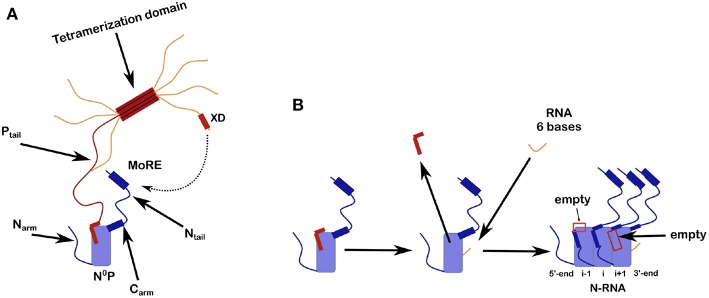
Scheme for N^0^P and the assembly of nucleocapsid-like particles (NCLP). **(A)** The N^0^P complex is made from one full N protomer with the N_arm_ and the C_arm_ that continues in the N_tail_ plus a tetramer of P. Close to the end of the N_tail_ there is the helical motif that could bind to the X domain of P. For this picture we show only one full P_tail_. The two N-terminal helices of P_tail_ bind N^0^ and for the crystal structures of N^0^P all the N_arm_, C_arm_ plus N_tail_ and almost all of P (only the first 2 helices of P are binding to N^0^) were cut away. **(B)** Scheme of the assembly of the NCLP with N^0^P with α_1/2_ of P and synthesized RNA (Milles et al., 2016). When the RNA binds N^0^, the two helices of P dissociates from the N^0^ and the monomer will assemble to the NCLP. In the NCLP, the N-terminal end of the N_arm_ forms a helix that binds to the protomer i-1 and the C_arm_ binds to protomer i+1. In the NCLP the 5′ and 3′ protomers both have one empty site.

N comprises two distinct parts: Folded N_core_ and N_tail_, an intrinsically disordered domain, containing a linear motif with α-helical propensity (residues 484–502; Figures 1, 2A). In solution this sequence folds into several helices of different length that exchange with each other and with a purely unfolded form on timescales faster than microseconds (Jensen et al., 2008, 2011; Communie et al., 2013b). This motif binds to the C-terminal domain of P, the X-domain (XD) (Figure 1; Johansson et al., 2003; Longhi et al., 2003; Kingston et al., 2004a,b; Bourhis et al., 2005; Houben et al., 2007; Schneider et al., 2015). Orthomyxoviruses like influenza virus have several separate RNA molecules, each bound to the viral polymerase. Because the nucleocapsids form circular complexes, with the 5′ and 3′ ends bound in close proximity on the polymerase (Ruigrok et al., 2011; Reich et al., 2014), the polymerase stays attached to the same gene while making new mRNAs. In paramyxoviruses such as measles, the polymerase first transcribes the leader RNA and then the mRNAs of N, P, M, F, H, and L. After each mRNA is terminated, L has to move from the end of the gene to the start of the next gene. The binding of N_tail_ and XD of P may assist in this process such that L remains in contact with the template (Brunel et al., 2014; Bloyet et al., 2016; Cox et al., 2017; Thakkar et al., 2018). The K_d_ of binding between N_tail_ and XD in paramyxoviruses is rather weak, between 3 and 8 μM (Schneider et al., 2015; Bloyet et al., 2016). If the affinity is artificially decreased further through mutations in the interacting protein domains, L has been shown to leave the template before transcribing all the genes (Bloyet et al., 2016).

N_core_ is flanked by N and C-arms which are disordered in the RNA-free form of the protein, and become more structured upon binding to the neighboring N_core_ subunits in nucleocapsids. During NC formation the N_arm_ and C_arm_ bind to the neighboring N protomers (Figure 2), with part of the N_arm_ forming a helix (residues 2–14) bound to the surface of the neighbor i-1 and the C_arm_ folding onto neighbor i+1. N can bind RNA, either viral RNA (vRNA) during infection, or cellular RNA during expression in cellular expression systems, making recombinant nucleocapsid-like structures. In order to avoid non-specific binding to cellular RNA, N is chaperoned by the N-terminal residues of P forming an N^0^P complex (Figures 1, 2A; Masters and Banerjee, 1988; Peluso and Moyer, 1988; Curran et al., 1993, 1995; Mavrakis et al., 2003). When the N_arm_ and C_arm_ from the N^0^P complex are removed, the complex can be crystallized and the structure can be determined through X-ray diffraction. In this way, the N^0^P structures of VSV, Nipah, measles, PIV5, HMPV, Marburg and Ebola viruses were determined (Leyrat et al., 2011; Yabukarski et al., 2014; Guryanov et al., 2015; Kirchdoerfer et al., 2015; Leung et al., 2015; Renner et al., 2016; Zhu et al., 2017; Aggarwal et al., 2018).

Up to now, there are no atomic resolution structures of the viral NCs, but NC-like particles (NCLPs) made from measles, Marburg, rabies and VSV viruses, for example, have similar structure as the viral nucleocapsids (Spehner et al., 1991; Fooks et al., 1993; Iseni et al., 1998; Mavrakis et al., 2002; Bhella et al., 2004; Schoehn et al., 2004). Some of the NCLPs assemble into rings rather than helical particles and the first two atomic resolution structures were determined from rings of VSV (10 protomers) and rabies (11 protomers per ring) with the RNA inside the ring (Albertini et al., 2006; Green et al., 2006). The N of RSV (respiratory syncytial virus) makes a ring with 10 to 11 protomers with the RNA on the outside of the ring (Tawar et al., 2009), the ring of PIV5 with 13 protomers with the RNA on the outside the ring (Alayyoubi et al., 2015) and HMPV with 10 protomers as with RSV (Renner et al., 2016). Most of the atomic-resolution structures were determined by crystallization and X-ray diffraction. Other NCLPs form helices of which the structures can be determined by Cryo-EM and single particle-based helical analysis. The first such structure was of measles virus NCLPs comprising 12.3 protomers (Gutsche et al., 2015) and Ebola virus with 24.4 protomers per turn of the helix and the RNA on the outside of the helix (Sugita et al., 2018), as with the rings of RSV and PIV5. Other structures of Ebola virus NCLPs with lower resolution show the same fold of the protein although the structure of the RNA was not visible (Wan et al., 2017; Su et al., 2018). A structure of the NCLP of mumps virus was also determined at a lower resolution (Cox et al., 2014; Severin et al., 2016). The comparison of N^0^P and the NCLPs structures reveals that, upon RNA binding the relative orientation of the lower and the upped lobes change by ~20^o^ (Green et al., 2006; Alayyoubi et al., 2015; Gutsche et al., 2015; Sugita et al., 2018) and became more closed than the N in the N^0^P from VSV, measles, PIV5 and Ebola viruses (Leyrat et al., 2011; Guryanov et al., 2015; Kirchdoerfer et al., 2015; Leung et al., 2015; Renner et al., 2016; Aggarwal et al., 2018) suggesting that in the N^0^P structures the RNA binding groove is primed for binding the RNA.

**Figure 3:**
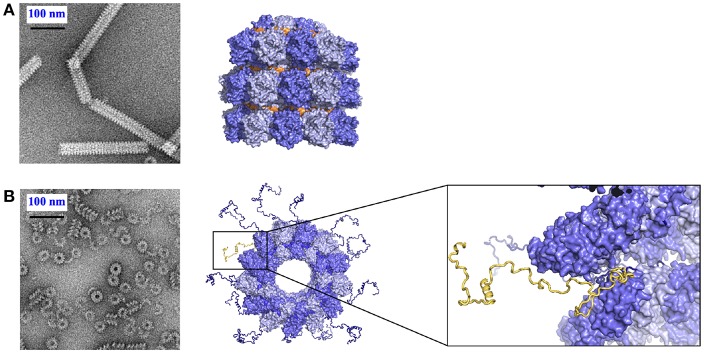
Role of N_tail_ in the nucleocapsid helix. **(A)** Tight helical NCLP with cleaved N with negative staining EM on the left and with the near-atomic structure on the right. The figure was made using PDB 4UFT. **(B)** Loose helical NCLP with full N with negative staining EM at left and with near-atomic structure with several tails. One N_tail_ with its helical motif is highlighted in yellow.

The structures of nucleoproteins of measles, PIV5, HMPV and Ebola viruses were determined in the absence of N_tail_. The NCs of measles virus are very flexible when N_tail_ is present, but form tighter helices when N_tail_ is removed, and these NCs can be used for higher resolution image analysis (Figure 3; Heggeness et al., 1980; Bhella et al., 2002, 2004; Schoehn et al., 2004; Renner et al., 2016). The structure of the NCLP of measles virus shows that the C_arm_ of N is close to the center of the helix (Desfosses et al., 2011; Gutsche et al., 2015) indicating that N_tail_ starts inside the helix. Solution state NMR showed that the first 50 residues of the tail are conformationally restricted before the chain escapes to the outside of the helix (Figure 3; Jensen et al., 2008, 2011). The disordered chain could interfere with stacking of turns, resulting in loosening of the helix, facilitating the functions of the transcription and replication from L. The helical linear motif is part of the C-terminal end of N_tail_ although N has been shown to function also when this helical motif was moved to different sites within N (Cox et al., 2017).

## Binding of the RNA by N

**Figure 4:**
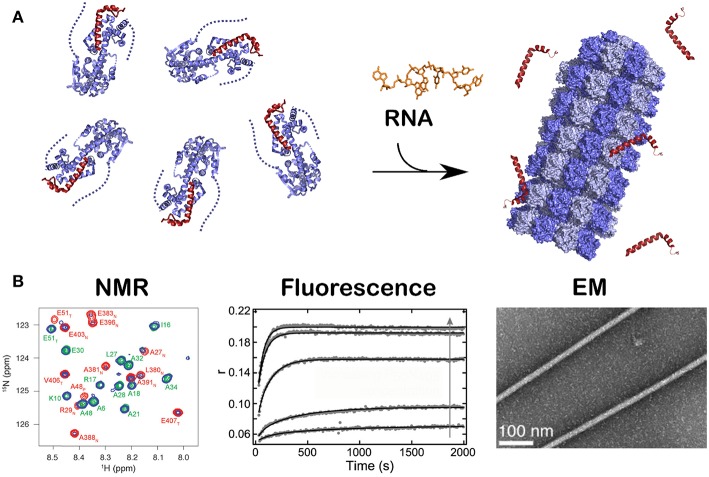
Kinetics of the assembly of NCLP by NMR, fluorescence and EM. Just like in Figure 2B, when RNA was given to the N^0^P complex, NCLP assembly could followed by NMR in real time because the NMR signals for the P peptide appear over time, by fluorescence anisotropy using fluorescein amidite-labeled RNAs, and by negative staining EM showing the nucleocapsids. For **(A)** we used PDB number 4CO6 for the N^0^P complex and PDB 4UFT for the NCLP, **(B)** is reprinted from Milles et al. (2016); Copyright 2016 Wiley-VCH Verlag GmbH Co. KGaA, used with permission.

**Figure 5:**
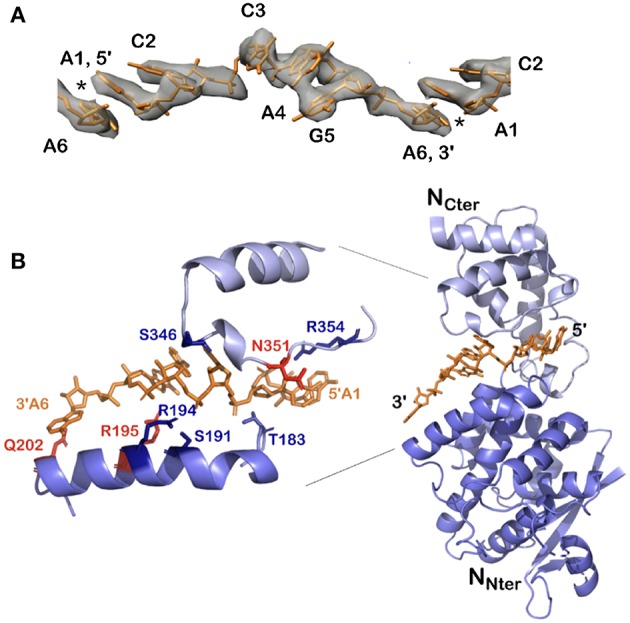
Structure of the 5′ viral genomic RNA (HO-ACCAGA-OH) inside the NCLP. **(A)** The Cryo-EM electrostatic potential of the 6 bases at the 5′ end of the viral RNA inside the NCLP (EM data base EMD-0142). The structures show gaps (asterisks) between bases 6 and 1, because the RNA (PDB 6H5S) molecules do not have a 5′ phosphate. **(B)** The protomer at the 3′ end inside the NCLP, showing that the last nucleotide is only bound to the base and not to the phosphate on the 2′ site on the ribose. The figure shows some of the residues binding the RNA, in red those that bind the bases. Reprinted with permission from Desfosses et al. (2019); Copyright 2019 National Academy of Sciences, used with permission. Frontier.

All existing paramyxoviral NCLPs structures were determined from recombinant protein bound to RNA from the cells in which N was expressed. This means that the RNA is probably of random sequence, and the structures have only been able to reveal binding of the phosphates and 2' oxygen of the ribose but not interactions involving sequence-specific bases. Milles and coworkers constructed a fusion N^0^P complex where the P peptide was fused on the N_arm_ with a TEV cleavage site (Milles et al., 2016). The purified complex was then cleaved to make the P peptide (residues 1–50) and either the full N (residues 1–525) or N up to the N_arm_ (residues 1–405), with no RNA bound (Figures 2B, 4). During incubation of this complex with synthesized RNA, the RNA binds, the P peptide comes off and N associates into NCLPs (Figure 2B). The kinetics of the assembly process can be studied by nuclear magnetic resonance (NMR) spectroscopy because the signal of P becomes narrow (peaks of P appear in the spectrum) when the assembly process is initiated and P peptide dissociates from N. The signal for the residues of N_arm_ and C_arm_ become broader (the peaks disappear) as they bind to the neighboring N particles in the NC. In addition, using RNA labeled with a fluorescent probe, the kinetics of the assembly can be followed by measuring the changes in fluorescence anisotropy. Free RNA tumbles rapidly, however once it is bound to N and then assembles into NCs, the anisotropy increases. The resulting assembly can then be visualized with negative staining EM and NC growth can be followed during several hours (Milles et al., 2016; Figure 4). The nucleoprotein from Sendai and measles viruses bind 6 RNA bases per protomer (Calain and Roux, 1993; Pelet et al., 1996) and micrometer long NCLPs could be made with RNA molecules of only 6 bases in length; either with the 5′ viral genomic RNA (HO-ACCAGA-OH) or a polyA (HO-AAAAAA-OH) sequence and the resulting NCLPs structures were determined with Cryo-EM and image analysis (Desfosses et al., 2019). The structures show the RNA with a gap between the hexamers because the RNA molecules do not contain a 5′ phosphate (Figure 5A). Whereas, the previous structure (Gutsche et al., 2015) mainly showed only N residues binding the sugar and the phosphate of the RNA (residues K180, T183, R194, R195, and R354) the new structures also show residues binding the RNA bases; R195, Q202, Y260, E263, N351 (Figure 5B; Desfosses et al., 2019).

**Figure 6:**
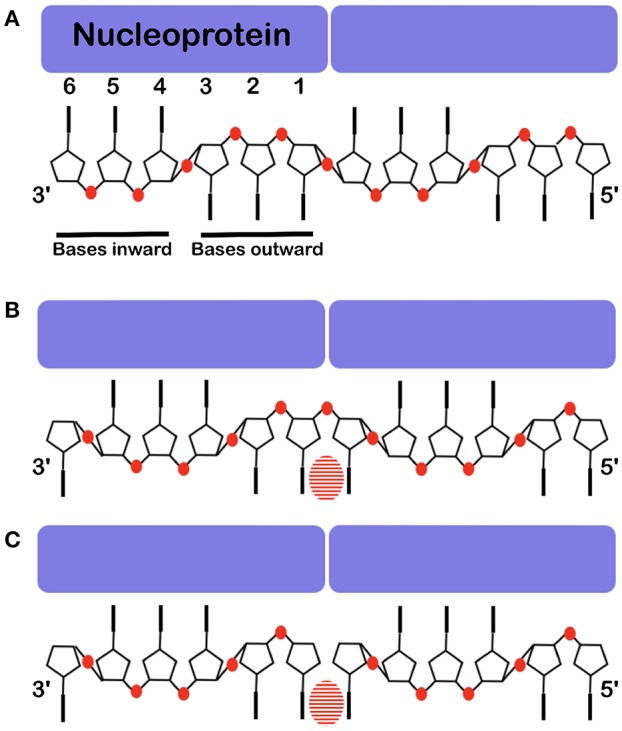
Base stacking over the interface between two protomers. The nucleoprotein binds 6 nucleotides, 3 point inward to the protein and 3 point outward. Often one cannot see clearly which base binds at the interface. **(A)** The structure of the RNA inside the NCLP of PIV5 (Alayyoubi et al., 2015). **(B)** The structure of the RNA inside the measles virus NCLP (Gutsche et al., 2015). **(C)** The structure of 6 nucleotides at the 5′ end of the viral RNA inside of the NCLP of measles virus (Desfosses et al., 2019). Because there is not a phosphate at the 5′, this shows the register of the RNA very clearly. The bases are shown, the phosphates are illustrated in red and the striped ovals show the stacking between two bases over the interface between two protomers.

The structures of VSV and rabies virus NCs show that each N protomer can bind 9 bases and RSV can bind 7 bases. The N protomer of measles, PIV5 and Ebola viruses bind 6 bases. In these nucleocapsids the RNA form 2 short A-helices connected by a conformational flip of the backbone RNA; all structures show that 3 bases turn to the nucleoprotein (inward) and the others turn outward (Figure 6). If the RNA followed a long A-helix rather than 2 short helices, then there would be no room for the nucleoprotein and, probably, the new RNA strand made by L during transcription could easily form a low energy double helix RNA structure. These viruses do not code for a helicase; so the nucleoprotein functions as a helicase (Iseni et al., 2000). For VSV/rabies and RSV, NCLP structures show that for each protomer, the last 3′ base stacks with the first 5′ base stabilizing the structure of the nucleocapsids between the two protomers (Albertini et al., 2006; Green et al., 2006; Tawar et al., 2009; Ruigrok et al., 2011). The structure of the NCLP of PIV5 suggests that nucleotides 4–6 turn inward and nucleotides 1–3 outward (3′-in-in-in-out-out-out-5′) and would mean that bases 6 and 1 do not stack (Figure 6A). However, the chemical modification of nucleotide bases in the Sendai viral nucleocapsids (Iseni et al., 2002) showed that bases 1 and 6 are both more open for modification than the other bases. The structure of the NCLP of measles virus (Gutsche et al., 2015) suggested that nucleotides turn 3′-out-in-in-in-out-out-5′. Nucleotides 6 and 1 stack just like the NCLP of rabies virus, VSV, and RSV (Ruigrok et al., 2011; Figure 6B). The structure of the synthesized NCLPs of measles virus with 6 nucleotides (Figure 6C) shows how the 6 nucleotides bind on the protomer and these structures show the same orientation of nucleotides as suggested for the previous structure, with stacking for nucleotides 6 and 1.

With the help of NCs formed *in vitro*, we can identify both the binding of the 5′ end of the RNA to the first N protomer of the NCs, and the binding of the 3′ end on the final protomer of the NCs (Figure 5B): The lower part of N is still bound to the RNA but the upper part of N does not cover the terminal bases and only residue Q202 in the lower edge of the groove binds the last base at the 3′ end. Q202 is important for replication initiation in PIV2 because the mutant Q202A shows increased replication (Matsumoto et al., 2017, 2018). The fact that the 3′ end of the RNA is rather “open” may help the binding of L at the 3′ end of the vRNA.

## Structure of the Disordered Phosphoprotein Tail, Residues 1–304

**Figure 7:**
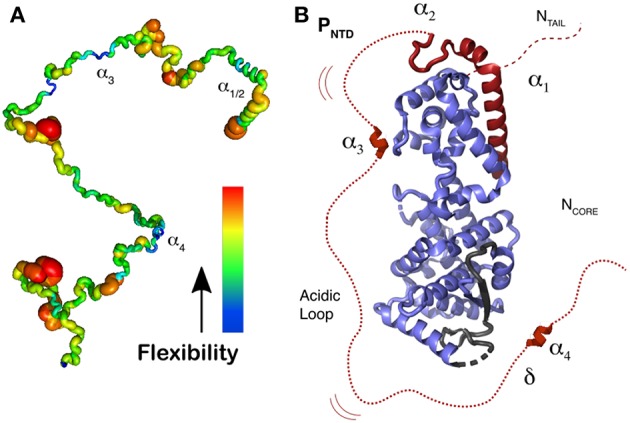
Representation of N^0^P with N^0^ and the 304 residues of P_tail_. **(A)** Localization of secondary structure and representation of flexibility/rigidity of P_tail_ with colors from red (flexible) to blue (rigid) and the transient α-helices from the N-terminal helix (α_1_ to α_4_). **(B)** Representation of N^0^P with the full P_tail_ showing α_1_ and α_2_ binding on N^0^, transient α_3_ and α_4_ region that weakly binds residues 96–127 of N^0^ as well the acidic loop between α_3_ and α_4_. For the N^0^ structure we used PDB 4CO6. Adapted from Milles et al. (2018); Copyright 2018 American Association for the Advancement of Science, used with permission. Frontiers.

NMR was used to investigate all the 304 disordered residues of the N-terminal domain of P, P_tail_, identify the structural behavior of each residue, and characterize the relative flexibility of P_tail_ (Jensen et al., 2011, 2014; Ozenne et al., 2012; Abyzov et al., 2016; Milles et al., 2016, 2018). Figure 7A shows the rigidity for the residues, with a thick red ribbon for the flexible regions and in thin blue ribbon for the more rigid regions, and 4 regions with a propensity for making helices. The first two helices represent the transient helices α_1/2_ that bind as helices in the N^0^P structure, residues 1–37, a short region around residue 110, α_3_, the STAT binding site (Devaux et al., 2007), and helix α_4_, around residues 189-198 (Milles et al., 2018). N^0^P with N^0^ and the 304 residues of P shows that α-helices 1 and 2 bind on N^0^ but also helix 4 and a short hydrophobic region just before helix 4, together named δα_4_ (delta-alpha-4). This region δα_4_ binds residues 96–127 on N^0^ (Figure 7B). This binding site and the helix α_4_ seem to be conserved in many paramyxoviral P proteins. If the “HELL” residues are changed to AAAA in a measles virus transcription/replication assay, the activity is suppressed (Milles et al., 2018). This means that the functional core of N^0^P is not only N^0^ plus helices 1 and 2 (Yabukarski et al., 2014; Guryanov et al., 2015) but also up to the δα_4_ region (Figure 7B). Between δα_4_ and α_3_ there is an acidic sequence with many Asp/Glu residues. As N^0^ cannot be purified in the absence of α_1/2_, it is complicated to measure the K_d_ for their binding to N^0^, but it is estimated to be in the nanomolar range as the complex can be purified on a size-exclusion column. The binding of δα_4_ to N^0^ is very weak, the K_d_ is around 0.6 mM, and we could show by NMR that δα_4_ binds and dissociates very rapidly while the N-terminal helices α_1/2_ remain bound, possibly so that the acidic loop blocks the cellular RNA binding to N^0^P.

The measles virus polymerase stutters on the template and adds extra G bases for making several mRNAs and therefore different proteins (Thomas et al., 1988; Cattaneo et al., 1989; Vidal et al., 1990). The V-protein uses the same P_tail_ as in the P-protein and changes sequence at residues 231 (the editing site, just after δα_4_) up to residue 299, to form a zinc-binding domain of the V protein (Cattaneo et al., 1989; Liston and Briedis, 1994). For P we know what the domains do: Residues 1-200 bind N^0^ and the rest of P_tail_ in another register forms part of V, 304-377 for the tetramerization domain and 457-507 for XD. We do not know the function of the region 378–456 (P_loop_) in the transcription and replication processes of measles virus. Additionally it should be mentioned that N and P are both known to be phosphorylated, which can be important for the viral cycle regulation, in particular phosphorylation is involved in the regulation of viral gene expression. However the mechanism of such regulation was not studied yet (Lamb et al., 1976; Sugita et al., 2018).

## Model for the Replication Complex of Measles Virus

**Figure 8:**
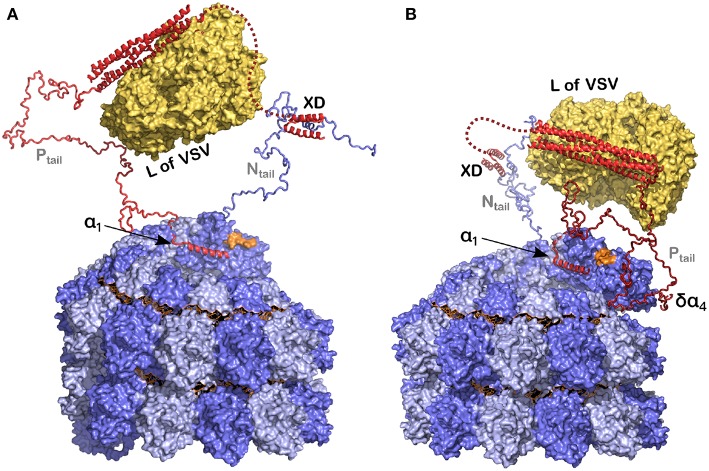
Model of the polymerase binding to the 3′ end of the RNA inside the NC. **(A)** The polymerase (L) binds to the tetramerization domain of P and P binds to the nucleocapsid, at the linear helical motif of N_tail_ with the XD, and potentially also with α_1_ of P on the empty site on the last protomer of the nucleocapsid. In orange is the final 3′ end of the viral RNA. **(B)** The same as in **(A)** but now with the δα_4_ region on residues 96–127 of N^0^ that may take the polymerase closer to the 3′ end of the RNA. For this figure we used PDB 3ZDO for the tetramerization domain of P, PDB 1T6O for the structure of the complex between the linear helical motif of N and the XD of P, the structure of the polymerase of VSV with PDB 5A22 and the NCLP of measles virus with PDB 6H5S. Modified from Desfosses et al. (2019); Copyright 2019 National Academy of Sciences, used with permission.

Cox and Plemper (2015) have suggested a model for the NCs in the presence of N, P and L for the paramyxoviruses. Similarly we have used recent results concerning the structures of P, N, and the nucleocapsids of measles virus (Figure 8) to develop a picture of the functioning polymerase complex. For this we used the structure of the NC with the 6 bases and for the structure of L we used the polymerase of VSV, to indicate the size of this component when bound to the tetramerization domain. In Figure 8 we show only one N_tail_ and only one P_tail_. P could be bound to N with the XD-N_tail_ complex and with α_1_ in the free α_1_-site on N (Figure 2) and L could act on the first bases of the 3′ end of the vRNA (Figure 8A). For the last base at the 3′ end, the 2' of the ribose and the phosphate would be free for binding L. The very long P_tail_ and N_tail_ could keep L away from the RNA. However, if δα_4_ region also binds to its binding site on N, then L could come closer to the vRNA (Figure 8B). To conclude, the current model illustrates the initial genomic RNA register and binding, and suggests a role for N-P interactions in positioning L during RNA processing.

## Author Contributions

RR and MB wrote the manuscript. SM and SG contributed to reviewing relevant literature, interpretation of studies, drafting the manuscript, and preparation of figures. All authors approved the article for publication.

### Conflict of Interest Statement

The authors declare that the research was conducted in the absence of any commercial or financial relationships that could be construed as a potential conflict of interest.
